# Isolated Myocysticercosis Diagnosed by Exclusion: A Case Report of Definitive Management With Surgery

**DOI:** 10.7759/cureus.81021

**Published:** 2025-03-22

**Authors:** Jyoti Sable, Pratik M Thakur, Archana Nehe, Jennifer Desai

**Affiliations:** 1 General Surgery, Bharatratna Dr. Babasaheb Ambedkar Hospital, Mumbai, IND

**Keywords:** intestinal cestode, myocysticercosis, parasitic tapeworm, taenia solium, tubular scolex

## Abstract

Cysticercosis, a parasitic ailment arising from the larval phase of *Taenia solium*, stands as a major health issue in the developing world. The encysted larvae can invade various body parts, with a higher prevalence in the brain, eye, skeletal muscle, and subcutaneous tissues. Isolated myocysticercosis is a rare entity and is often found to be associated with the involvement of the central nervous system. Nonetheless, we report a case of isolated myocysticercosis of the gastrocnemius muscle devoid of any systemic implications. The purpose of this case report is to consider myocysticercosis as a differential diagnosis in cases of soft tissue swellings in both vegetarian and mixed-diet individuals, as well as to discuss the available treatment options and prevention methods.

## Introduction

Human cysticercosis is caused by a parasite called *Taenia solium*, also known as the pork tapeworm. In developing countries, factors such as rural communities, overcrowding, and poor sanitation contribute to increased human-pig interaction and a higher risk of fecal-oral contamination, making tapeworm infections prevalent [1]. This infection is now also observed in developed nations due to increased migration. Cysticercosis can occur in both vegetarian and non-vegetarian humans. Food handlers who do not practice hygiene by washing their hands are responsible for contamination while working. Fruits and vegetables can also be contaminated by fertilized parasites [2]. Poor sanitation leading to environmental fecal contamination is a major factor in transmission. Cysticercosis mainly affects low- and middle-income countries in Africa, Asia (e.g., India, China, and Nepal), and Latin America (e.g., Guatemala, Nicaragua, El Salvador). According to Bhardwaj et al., "the larval cyst of human cysticercosis occurs in order of frequency in the brain, uterus, vitreous humor of the eye, striated muscle, and subcutaneous tissues. Isolated muscular involvement is uncommon. Till date, only a few cases of muscular cysticercosis have been reported" [3]. 

The life cycle of* Taenia solium* involves two hosts: (i) the definitive host: humans, which harbor the adult worm; (ii) the intermediate host: pigs, which harbor the larval stage. The adult worm lives in the small intestine (upper jejunum) of humans. The eggs or gravid segments are passed out with the feces onto the ground. Pigs swallow these eggs by drinking contaminated water or by eating uncooked vegetables infected with eggs. Upon reaching the alimentary canal of the intermediate host, the radially striated walls of the eggs rupture, releasing oncospheres. These oncospheres penetrate the gut wall with the aid of their hooks and enter the portal vessels or mesenteric lymphatics, ultimately reaching the systemic circulation. Typically, they travel via the portal vein and successively reach the following organs: the liver, the right side of the heart, the lungs, the left side of the heart, and the systemic circulation. The naked oncospheres are filtered out from the circulating blood into the muscular tissues where they ultimately settle and undergo further development. The oncospheres lose their hooks upon reaching their destination, the cells in the center liquefy, and about eight days after infection, each oncosphere forms an oval vesicle that gradually increases in size, containing at its bottom the larva (the scolex or the future "head" of the adult worm) referred to as cysticercus. It takes about 60 to 70 days for the oncospheres to metamorphose into the cysticercus stage. Humans become infected by eating undercooked pork that contains cysticerci. Inside the human alimentary canal, the scolex, upon contacting bile, exvaginates and anchors to the gut wall by means of its suckers, gradually developing into an adult worm through strobilation. Humans may occasionally serve as the larval host of *Taenia solium*, becoming infected in the same way as pigs, either by drinking contaminated water or by eating uncooked vegetables infected with eggs. Additionally, a person harboring the adult worm may auto-infect themselves due to unclean and unhygienic personal habits. The clinical features of cysticercus cellulosae infection may vary. It can be asymptomatic or symptomatic. Neurocysticercosis and cysticercus cellulosae in the eyes are two of the most common presentations of the disease. 

## Case presentation

**Figure 1:**
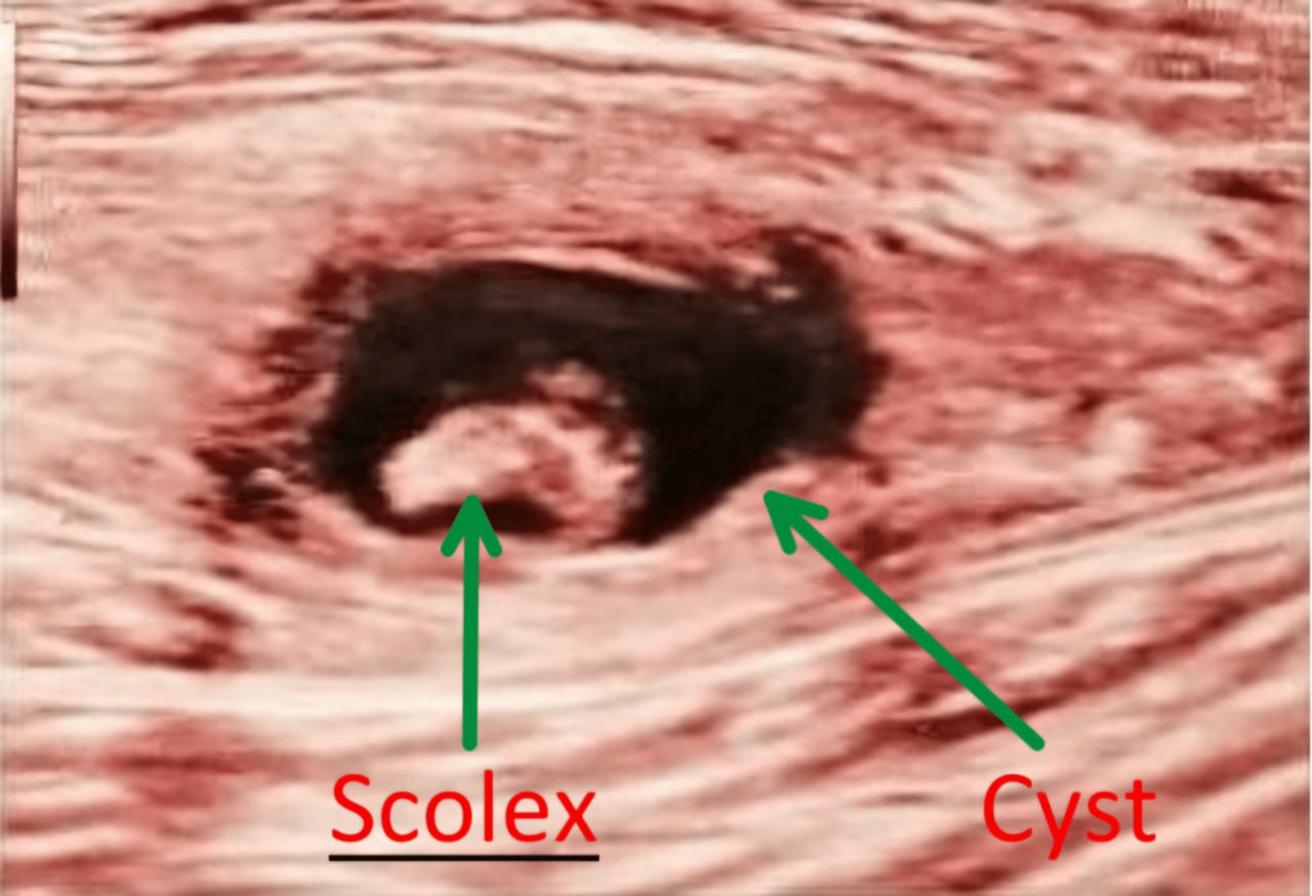
Preoperative ultrasound image Ultrasonography of the right calf revealed scolex of *Taenia solium *characterized by an eccentric echogenic mass surrounded by a hypoechoic fluid-filled cyst.

A 32-year-old vegetarian male patient who works as a garment merchant near his home presented with pain and a lump in his right calf for the past six months. This patient consumes tap water directly without sterilization methods such as boiling, which is supplied by the municipal corporation. He does not eat outside food and primarily consumes meals prepared by his family. He reported no history of trauma, leg swelling, signs of inflammation, sensory or motor deficits in the affected leg, epilepsy, or other constitutional symptoms. Examination revealed a tender, firm, almond-shaped swelling measuring 3.0 × 2.0 cm in the right calf region. The swelling had a smooth surface and vague margins, and it was embedded within the tissue, not attached to the skin. Clinically, a differential diagnosis of neurofibroma, lipoma, pyomyositis, and epidermoid cyst was considered. Ultrasonography showed a thick-walled cystic lesion of 1.9 x 1.0 x 1.7 cm in the medial head of the gastrocnemius muscle, with a 5 mm tubular scolex and surrounding edema suggestive of myocysticercosis (Figure 1). Stool routine and microscopy were negative for any worms or eggs.

**Figure 2:**
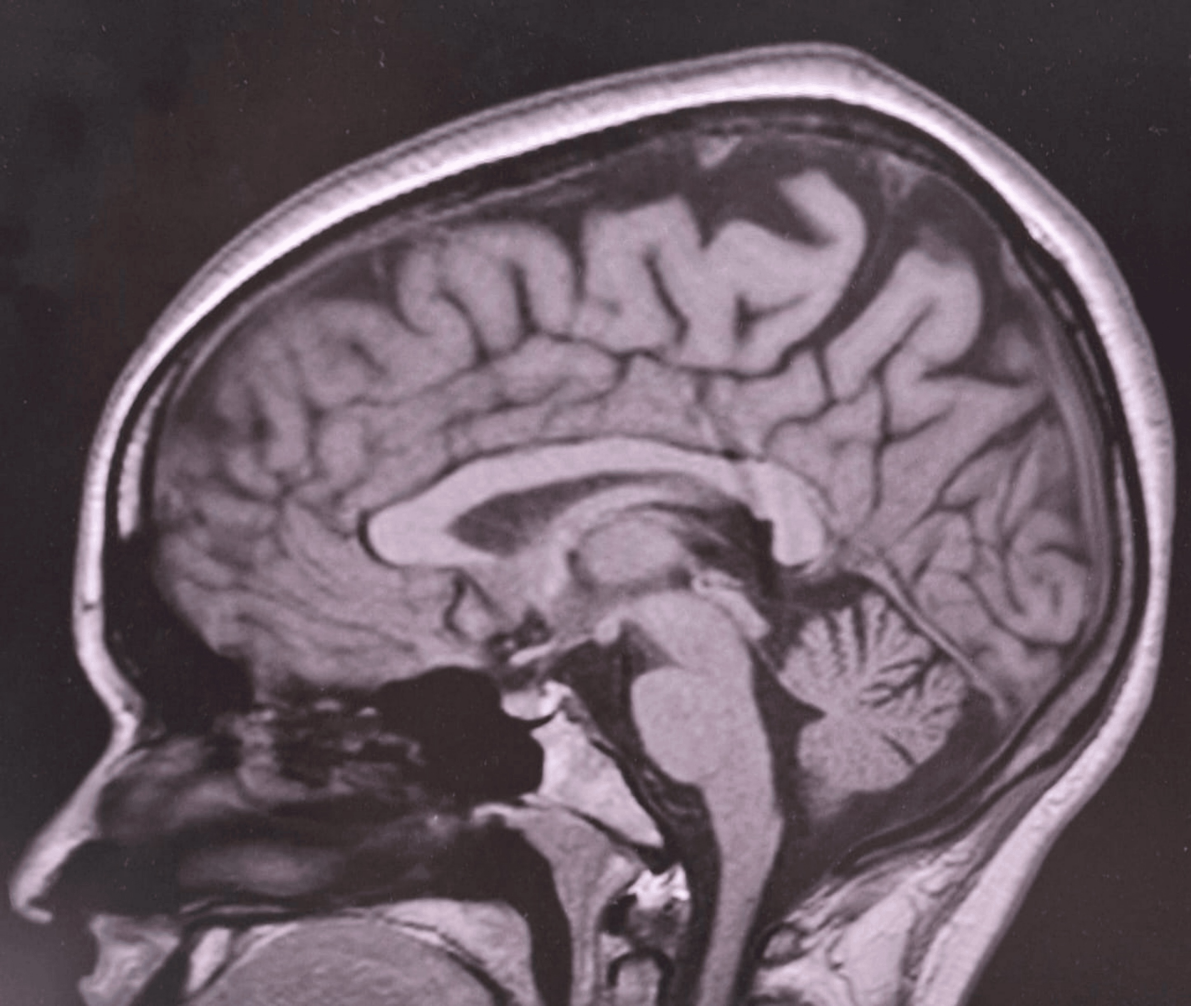
MRI of the patient's brain The brain MRI did not show any well-defined round lesion, which is characteristic of neurocysticercosis.

**Figure 3:**
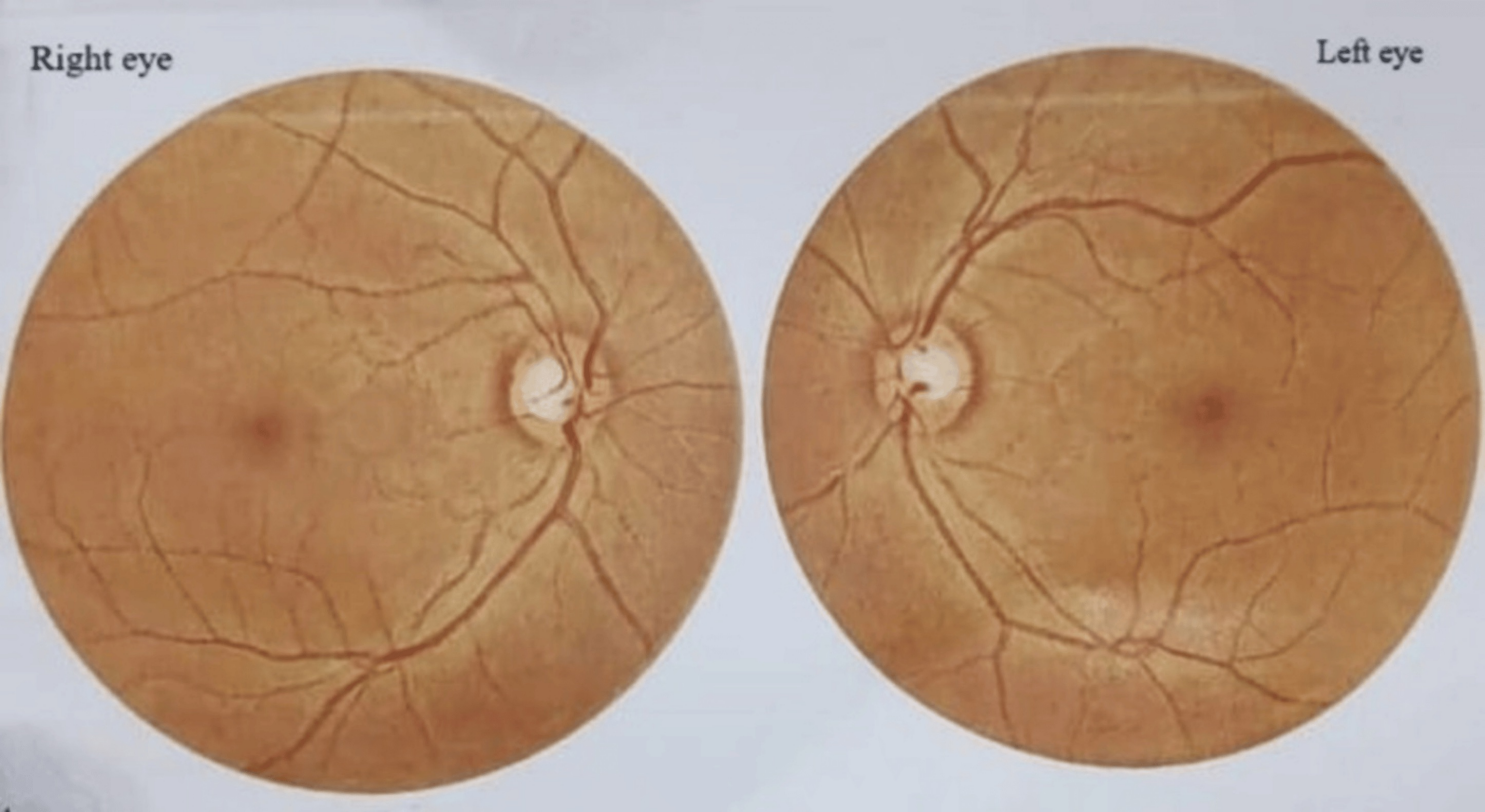
Indirect ophthalmoscopy picture In indirect ophthalmoscopy, a normal posterior segment (vitreous humor and retina) was observed.

**Figure 4:**
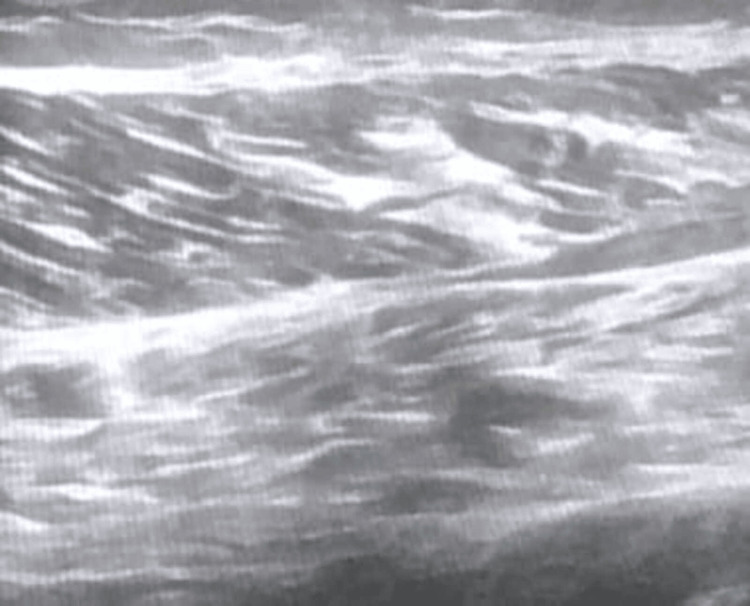
Postoperative ultrasonography image of the calf region The previously seen cyst with scolex was not observed in the postoperative ultrasonography picture.

MRI of the brain (Figure 2) and indirect ophthalmoscopy (Figure 3) were both normal, leading to a diagnosis of isolated myocysticercosis. The patient was started on albendazole 400 mg twice a day, but his pain increased after a week, prompting surgical excision, after which he responded well. The cyst was completely excised without spillage. Postoperatively, the patient received albendazole for three more weeks. A repeat ultrasound showed postoperative changes and no focal lesion (Figure 4).

**Figure 5:**
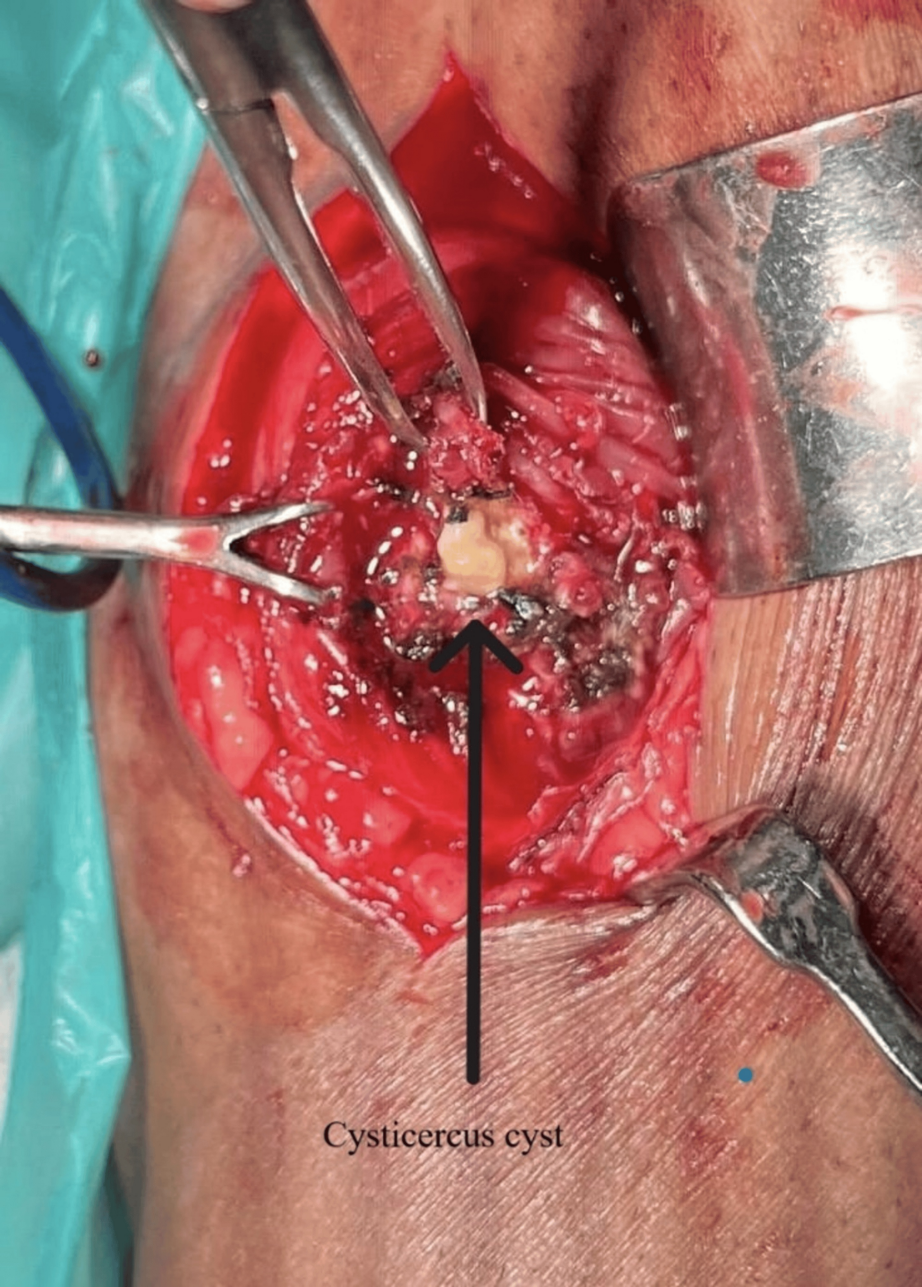
Intraoperative finding An intraoperative picture of the calf muscle showed a cysticercus cyst.

Intraoperative photographs of isolated myocysticercosis revealed a cyst approximately 2 x 2 cm (marked by a black arrow in Figure 5) in the medial head of the gastrocnemius muscle, surrounded by fibrosis. The postoperative histopathology report was inconclusive regarding any specific pathology. An anti-cysticercal IgG antibody test was conducted during the postoperative period using the enzyme-linked immunosorbent assay (ELISA) method, which returned negative with an observed value of 4.69 NTU. It is defined as positive if greater than or equal to 11 NTU.

## Discussion

According to Minhas et al., taeniasis and cysticercosis can be eliminated through preventive measures such as well-cooked pork and washing vegetables, respectively [1]. Cysticercosis is caused by ingesting food and water infected with embryonated *Taenia solium *eggs, while taeniasis results from consuming undercooked meat of the intermediate host, like pork. Humans are the definitive host, and pigs are the intermediate host. Cysticercosis most commonly affects the brain and eyes, and isolated muscle involvement is rare. Therefore, it is important to perform an MRI of the brain and indirect ophthalmoscopy to rule out nervous and ocular cysticercosis. High-definition ultrasound is adequate for diagnosing myocysticercosis [4]. CT scans and MRIs are useful in assessing the relationship with surrounding structures like major nerves and vessels. MRI can be used for disseminated myocysticercosis and is superior to CT scans in evaluating and detecting the stage of the disease, as well as in revealing perilesional edema. MRI can show live scolices and cysts, along with degenerating cysts. Additionally, preoperative fine needle aspiration cytology (FNAC) can be a rapid and useful modality. In FNAC, it presents as a collection of eosinophils, neutrophils, histiocytes, or granuloma formation. FNAC samples may also show fragments of larvae, hooklets, palisading histiocytes, granuloma, and mixed inflammation. However, sensitivity is low since the aspirated sample may not be representative of the disease. 

Newer immunological modalities such as enzyme-linked immunoelectrotransfer blot (EITB) and ELISA are effective in demonstrating anti-cysticercus antibodies. According to the Centers for Disease Control and Prevention (CDC) Yellow Book, results in the EITB test can be negative in over 30% of patients with a single parenchymal lesion [5]. Gupta et al. describe three types of clinical manifestations of muscular cysticercosis: "the myopathic type; the mass-like type; and the pseudo-hypertrophy type, in which multilocular cysts form in a group of muscles" [6]. Our patient exhibited both myalgic and mass-like types. Additionally, four different types of sonographic appearances of cysticercosis have been described: (i) cysticercus cyst with an inflammatory mass surrounding it due to larval death (this picture is seen in our case); (ii) cysticercus cyst with fluid leakage from one side; (iii) an irregular large collection of exudative fluid in the muscle with a typical cysticercus cyst and an eccentric scolex in the collection; (iv) a calcified cyst appearing as multiple elliptical calcifications in soft tissue, giving a millet seed appearance on X-ray [7].

The postoperative histopathology report was inconclusive, possibly due to the antihelminthic medication (albendazole) received in the preoperative period, which causes degenerative changes in cysticercus cysts. Solitary uncomplicated myocysticercosis is managed conservatively with tab albendazole 15 mg/kg/day in two divided doses for 4-6 weeks or tab praziquantel. Steroids are usually not necessary for such cases. Steroids are indicated in disseminated cysticercosis and neurocysticercosis to suppress the inflammatory reaction induced by the release of dying parasitic antigens. However, in myocysticercosis, only antihelminthic therapy is sufficient [8]. Isolated muscular cysticercosis may require surgical excision when it is painful and pressure symptoms appear or when it is unresponsive to albendazole [9]. In cases where patients are unresponsive and do not wish to undergo surgical management for reasons such as financial constraints or domestic circumstances, they can opt for percutaneous aspiration, injection, re-aspiration (PAIR) treatment with 3% hypertonic saline. PAIR, when combined with albendazole, yields good results [10].

## Conclusions

Isolated myocysticercosis is a rare presentation of cysticercosis. It can affect both vegetarians and individuals on mixed diets. In endemic countries like India, water reservoir plants and supply pipelines should be inspected for contamination by human sewage, which can occur due to human settlements near water supply pipelines in the form of slums. These slums do not have proper sewage disposal systems, and residents procure water for consumption from the main water supply pipeline through illegal and unauthorized pipe connections, which leads to contamination of potable water. Therefore, in these endemic areas, water should be consumed after sterilization procedures, such as boiling tap water.

In patients with swelling in the intramuscular plane, a differential diagnosis of cysticercosis should be considered. Ultrasonography is usually the first investigation of choice for swelling in the body. Myocysticercosis can be diagnosed with the assistance of ultrasonography. Preoperative ultrasound-guided FNAC is a simple investigation that may help demonstrate the parasite. Serological investigations, such as anti-cysticercus IgG antibodies, can serve as useful adjuncts to clinical and radiological diagnosis for myocysticercosis. Once myocysticercosis is confirmed, neurological and ocular cysticercosis should be ruled out through brain imaging and indirect ophthalmoscopy. Uncomplicated solitary myocysticercosis can be managed conservatively with albendazole. Only complicated cases with abscesses, nerve compression, and painful lesions that do not respond to medication warrant surgery. Steroids are generally not needed for isolated cases due to the very low parasitic load. 
